# CAN PRODUCTIVE AGING BE MADE TO WORK FOR ALL WORKERS?

**DOI:** 10.1093/geroni/igz038.1370

**Published:** 2019-11-08

**Authors:** Philip Taylor

**Affiliations:** Federation University Australia, Melbourne, Victoria, Australia

## Abstract

This paper considers the changing status of older workers in Organisation for Economic Co-operation and Development (OECD) countries and addresses the questions of if and how they can be supported to age productively. This paper questions the utility of the dominant pro-work policy framework which, while ostensibly aimed at all older workers, mostly benefits those for whom greater choice around work and retirement was always available. Analysis of OECD data concerned with employment and unemployment demonstrates that, for a significant proportion of older workers, choice in terms of labor force participation is severely constrained, with potentially adverse consequences for the transition to old age. The soundness of the pro-work agenda is challenged and it is argued that older people’s advocacy, in particular, has an important role to play in offering a vision of what it means to grow old successfully that is not limited by narrow conceptions of productivity.

